# News and Notes

**Published:** 1908-09

**Authors:** 


### NEWS AND NOTES

Dr. and Mrs. W. C. Jarnagin have been spending the summer at Norcross.

Dr. J. L. Campbell and family recently enjoyed a very pleasant stay at Tallulah Falls.

Dr. and Mrs. G. P. Huguley have returned from a very pleasant trip to North Carolina.

Dr. J. Dawkins Cromer spent several days recently at Toxaway.

Dr. A. W. Calhoun's many friends will be glad to learn that he has sufficiently recovered to be able to leave Johns Hopkins, where he has been ill for some time.

Dr. A. L. Lawton spent several days in Atlanta recently. Dr. Lawton is now resident physician to Toxaway Hotel.

Dr. and Mrs. Floyd W. McRae spent a portion of August in New York.

Dr. Chas. R. Andrews enjoyed a very pleasant stay and delightful fishing in the mountains of North Georgia during the past month.

The many friends of Dr. E. C. Ripley will regret to learn that he has removed his residence to Meriwether county, where he will continue in the practice of medicine.

---

The Seventh District Society of Physicians and Surgeons will convene at Cartersville, Ga., on October 14th. An interesting program is anticipated.

---

In the death of Dr. H. B. McMaster, which occurred on the 21st, not only the medical profession, but the state at large, has suffered a severe loss. The community of Waynesboro, in particular, will miss the services of this devoted and conscientious worker.

---

Dr. M. B. Hutchins spent several days in the mountains of North Carolina and reports a most enjoyable vacation.

---

Dr. H. M. Smith, of Edgewood, Ga., left recently to visit his son, Dr. Leonard Smith, who is ill at Clayton, Ga.

---

Dr. J. Edgar Paullin spent several weeks recently in and about Fort Gaines and Marshallville.

---

In 1907 the mortality from plague in India reached the appalling total of 1,204,194. But a rapid decline has now occurred and the number of deaths in the twelve months ending June 30, 1908, is only 151,781.

---

Mrs. Emanuel J. and William N. Senn have presented to the John Crerar Library, of Chicago, more than 2,000 volumes from the library of the late Dr. Nicholas Senn, to be added to the Senn collection.

---

The names of 189 physicians have been stricken from the register of physicians by the Ontario Medical Council on account of non-payment of dues for three or more years. These men have declined, on principle, to pay the dues, holding that when they once become qualified physicians they should not be compelled to pay annual charges.

Dr. William Osler, Oxford University, has been made lord rector of Edinburg University.

---

Dr. and Mrs. James B. Baird enjoyed quite an extensive trip through Canada, stopping at Atlantic City and other resorts on their way South.

---

The annual meeting of the British Medical Association was held at Sheffield on July 27-31, and was attended by nearly 1,000 members.

---

By the will of Frederick Hewett Owego, $2,000,000 is bequeathed to the New York Post-Graduate School and Hospital.

---

Dr. Roy Van Wart, of New Orleans, Assistant Neurologist of Tulane University, was recently in Atlanta visiting his mother at Howell Park Sanatorium.

---

The Scottish local government report just issued shows that a very energetic campaign against consumption is being waged by the authorities of that country.

---

Dr. Lewis M. Gaines, who is on the staff of the Journal-Record, announces elsewhere in this issue that he will give quizzes to aid those preparing to stand State Board and other medical examinations.

Dr. Gaines has had unusual advantages to equip him for this kind of work, having taught anatomy and physiology in one of the leading medical colleges of North Carolina for three years, and the coming session will give the course in materia medica at the Atlanta Schiil of Medicine.

---

For the first time in several years in this city the degree of Master of Pharmacy was conferred upon five distinguished men from different sections of the United States, who have attained distinction in the art of preparing medicines and drugs. Those who received this honor were Samuel W. Fairchild, of New York; Horatio elson Fraser, of New York; John F. Hancock, of Baltimore; S. A. D. Sheppard, of Boston, and William McIntyre, of Philadelphia.—*Evening Bulletin, Philadelphia.*

At the annual meeting of the faculty of the Mississippi Medical College, Dr. William W. Hamilton was elected president; Dr. Nathan L. Clark, vice-president; Dr. Samuel H. Mairston, secretary; O. W. Bethea, treasurer; and Dr. T. Alexander Brown, dean.

---

The Elkin-Goldsmith Sanatorium has recently issued the fifth biennial report of the surgical cases treated during the past two years in this well known institution. The report makes an excellent showing and demonstrates the high grade of surgery being done in Atlanta.

